# Supplementary material for: Entropic and Near-Field Improvements of Thermoradiative Cells
Source: Sci Rep. 2016 Oct 13;6:34837. doi: 10.1038/srep34837 (PMC5062074; doi:10.1038/srep34837)
Supplement: Supplementary Information [file srep34837-s1.pdf]

Supplementary Material

Entropic and Near-Field Improvements of Thermoradiative Cells

Wei-Chun Hsu, Jonathan K. Tong, Bolin Liao, Yi Huang, Svetlana V. Boriskina,[a)] and Gang Chen[b)]

Department of Mechanical Engineering, Massachusetts Institute of Technology, Cambridge, Massachusetts 02139, USA

Corresponding Authors

a)E-mail: sborisk@mit.edu (S. V. Boriskina)

b)E-mail: gchen2@mit.edu (G. Chen)

Section I. The Thickness Dependence of the Thermoradiative Cells

The performance of the thermoradiative cells depend on the thickness of the InSb thin film. Both efficiencies and power densities are calculated for three configuration including (i) a thin-film InSb, (ii) a thin-film InSb and a selective surface of $\Delta\hbar\omega = 0.01$ eV, and (iii) a thin-film InSb and a bulk $CaCO_3$ at a gap distance of 10nm. The optical constants of InSb and $CaCO_3$ used in all the calculations are also shown in Fig. S1 [1-3].

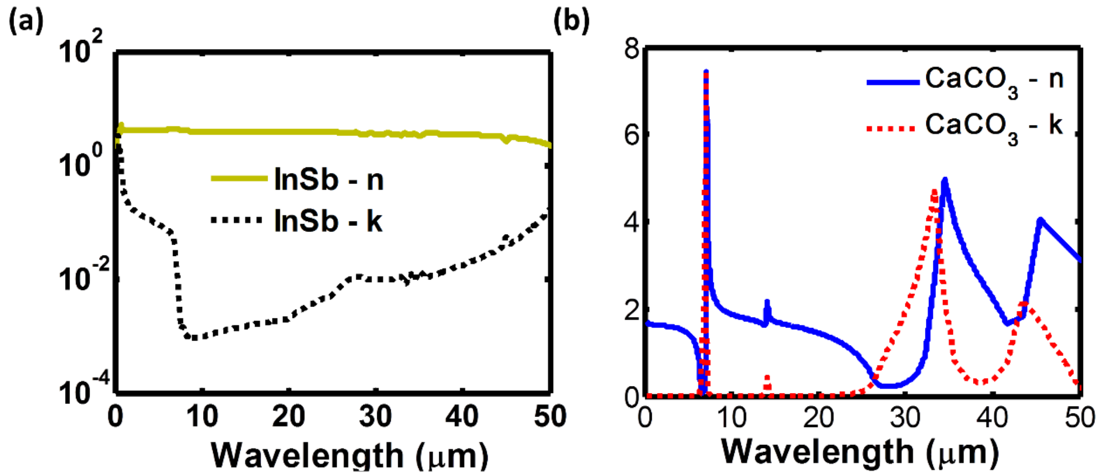

Figure S1: Optical parameters of materials used in the calculations of the thermoradiative cell. (a) Indium antimonide (InSb). (b) Calcium carbonate ($CaCO_3$). n is the real part of the refractive index. k is the imaginary part of the refractive index.

For each configuration, the ideal case and the other two cases that take into account the sub-bandgap and non-radiative losses are calculated and shown in Figs. S2, S3, and S4. The ideal cases (red solid curve) only include the radiative process, which contributes to the emission where the photons have energy higher than the bandgap energy, to create the negative chemical potential and generate the power. For the real material, the emission include the photons with their energy below the bandgap energy. These photons cannot contribute to the formation of chemical potential and result in the sub-bandgap loss (blue solid curve). However, there are also non-radiative processes such as Auger, Shockley-Read-Hall, and surface defect processes. These processes will result in the net charge generation rate when the chemical potential is

negative, and the net generation will reduce the magnitude of chemical potential and cause the electronic loss or non-radiative loss (black solid curve).

There are several important points for each case. First, the efficiency is higher for thinner thermoradiative cells, especially for the configuration (iii) that relies on the near-field radiative transfer; however, power density increases with thickness for each configuration except for the near-field radiative transfer including both sub-bandgap and non-radiative losses (blue data shown in Fig. S4). Second, if the non-radiative rate can be significantly reduced, the efficiency is higher if a selective suface is used to control the emission. If the non-radiative rate is comparable with the radiative rate, the case using near-field radiative transfer has the highest efficiency due to its enhanced radiative rate. Third, there are some oscillations of efficiencies and power densities for various thicknesses, as shown in the Figs. S2 and S3. They happen due to the interference inside the InSb thin film resulting in the 'thermal well' effect. [4-5]

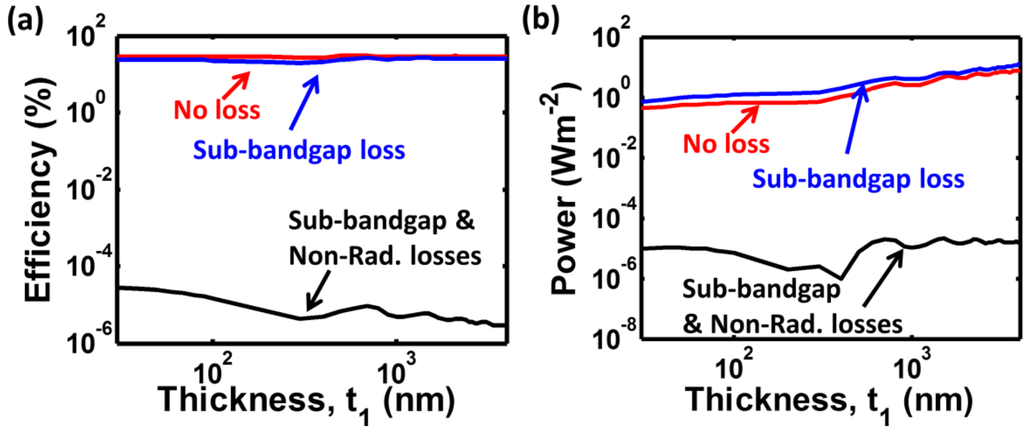

Figure S2: The performance of the thermoradiative cells for various thicknesses of the thin-film InSb. The air is at 300K. (a) Efficiency (b) Power density.

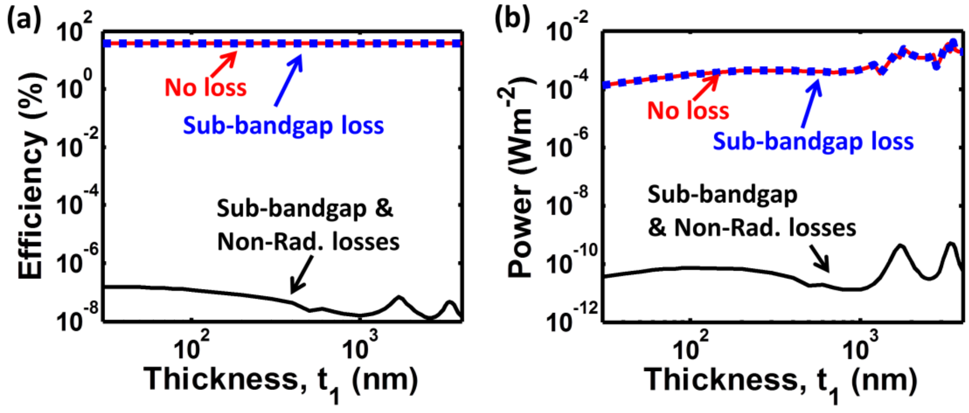

Figure S3: The performance of the thermoradiative cells for various thicknesses of the thin-film InSb with a selective surface of $\Delta\hbar\omega = 0.01$ eV. The air is at 300K. (a) Efficiency (b) Power density.

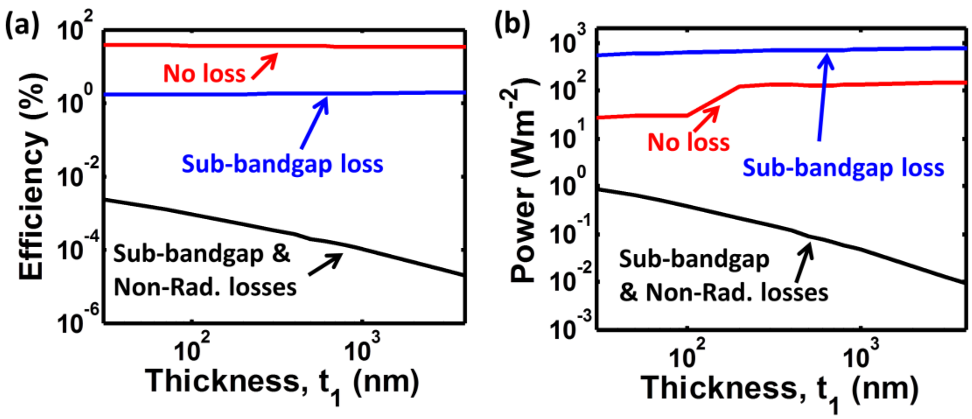

Figure S4: The performance of the thermoradiative cells for various thicknesses of the thin-film InSb, combining with a bulk $CaCO_3$ at the gap distance of 10nm. Cold sink, $CaCO_3$, is at 300K (a) Efficiency (b) Power density.

Section II. The Formulas for the Near-Field Thermal Radiation Between Two Thin Films.

To calculate the near-field thermal radiation between a thin film and a semi-infinite substrate, we use the formulas that are derived from references 1 and 2. The Planck oscillator term, $\theta(\omega,T)$, determines the thermal distribution of modes contributing to the radiative heat transfer and in its most general form includes the Bose-Einstein distribution and a zero-point energy term as follows,

$$\theta(\omega,T)=\frac{\hbar\omega}{2}+\frac{\hbar\omega}{e^{\frac{\hbar\omega}{k_BT}}-1} \tag{S1}$$

where $\hbar$ is the reduced Planck constant, k_B is the Boltzmann constant, ω is the frequency of the photon, and T is temperature of the emissive body. For transport calculations, the zero-point energy term is negated by emission from both the hot and cold media.

For TM and TE polarizations, respectively, the Fresnel coefficients for an interface are,

$$r_{ij}^{TM}=\frac{\varepsilon_j k_{z,i}-\varepsilon_i k_{z,j}}{\varepsilon_j k_{z,i}+\varepsilon_i k_{z,j}} \qquad t_{ij}^{TM}=\frac{2k_{z,i}\sqrt{\varepsilon_i}\sqrt{\varepsilon_j}}{\varepsilon_j k_{z,i}+\varepsilon_i k_{z,j}} \tag{S2}$$

$$r_{ij}^{TE}=\frac{k_{z,i}-k_{z,j}}{k_{z,i}+k_{z,j}} \qquad t_{ij}^{TE}=\frac{2k_{z,i}}{k_{z,i}+k_{z,j}} \tag{S3}$$

where ε_i and ε_j is the complex dielectric permittivity for layers i and j, respectively. Likewise, $k_{z,i}$ and $k_{z,j}$ is the perpendicular wavevector for layers i and j. For a thin slab with index j, the reflection and transmission coefficients are,

$$r_{i\ell}^{TM,TE}=\frac{r_{ij}^{TM,TE}+r_{j\ell}^{TM,TE}e^{2ik_{z,j}d_j}}{1+r_{ij}^{TM,TE}r_{j\ell}^{TM,TE}e^{2ik_{z,j}d_j}} \qquad t_{i\ell}^{TM,TE}=\frac{t_{ij}^{TM,TE}t_{j\ell}^{TM,TE}e^{ik_{z,j}d_j}}{1+r_{ij}^{TM,TE}r_{j\ell}^{TM,TE}e^{2ik_{z,j}d_j}} \tag{S4}$$

where d_j is the thickness of the layer j.

To isolate the radiative heat flux from film 1 to film 3, a simple subtraction is needed using equations (S2) and (S3) as follows,

$$q_{\omega,prop}^{1\to 3}\left(\omega,T_1\right)=\frac{\theta\left(\omega,T_1\right)}{\pi^2}\int_0^{k_v}k_r dk_r\left\{\frac{\left[1-\left|r_{20}^{TE}\right|^2-\left|t_{20}^{TE}\right|^2\cdot\mathrm{Re}\left(\frac{k_{z,0}^{*}k_{z,2}^{'}}{\left|k_{z,2}\right|^2}\right)\right]\cdot\left[1-\left|r_{24}^{TE}\right|^2-\left|t_{24}^{TE}\right|^2\cdot\mathrm{Re}\left(\frac{k_{z,4}^{*}k_{z,2}^{'}}{\left|k_{z,4}\right|^2}\right)\right]}{4\left|1-r_{20}^{TE}r_{24}^{TE}e^{2ik_{z,2}^{'}g}\right|^2}+\ldots\right.$$

$$\left.\ldots+\frac{\left[1-\left|r_{20}^{TM}\right|^2-\left|t_{20}^{TM}\right|^2\cdot\mathrm{Re}\left(\frac{k_{z,0}k_0^{*}k_{z,2}^{'}}{k_0\left|k_{z,2}\right|^2}\right)\right]\cdot\left[1-\left|r_{24}^{TM}\right|^2-\left|t_{24}^{TM}\right|^2\cdot\mathrm{Re}\left(\frac{k_{z,4}k_4^{*}k_{z,2}^{'}}{k_4\left|k_{z,2}\right|^2}\right)\right]}{4\left|1-r_{20}^{TM}r_{24}^{TM}e^{2ik_{z,2}^{'}g}\right|^2}\right\} \qquad \text{(S5)}$$

$$q_{\omega,eva}^{1\to 3}\left(\omega,T_1\right)=\frac{\theta\left(\omega,T_1\right)}{\pi^2}\int_{k_v}^{\infty}k_r dk_r e^{-2k_{z,2}^{''}g}\left\{\frac{\left[\mathrm{Im}\left(r_{20}^{TE}\right)-\frac{\left|t_{20}^{TE}\right|^2}{2}\cdot\mathrm{Re}\left(\frac{k_{z,0}^{*}k_{z,2}^{''}}{\left|k_{z,2}\right|^2}\right)\right]\cdot\left[\mathrm{Im}\left(r_{24}^{TE}\right)-\frac{\left|t_{24}^{TE}\right|^2}{2}\cdot\mathrm{Re}\left(\frac{k_{z,4}^{*}k_{z,2}^{''}}{\left|k_{z,2}\right|^2}\right)\right]}{\left|1-r_{20}^{TE}r_{24}^{TE}e^{-2k_{z,2}^{''}g}\right|^2}+\ldots\right.$$

$$\left.\ldots+\frac{\left[\mathrm{Im}\left(r_{20}^{TM}\right)-\frac{\left|t_{20}^{TM}\right|^2}{2}\cdot\mathrm{Re}\left(\frac{k_{z,0}k_0^{*}k_{z,2}^{''}}{k_0\left|k_{z,2}\right|^2}\right)\right]\cdot\left[\mathrm{Im}\left(r_{24}^{TM}\right)-\frac{\left|t_{24}^{TM}\right|^2}{2}\cdot\mathrm{Re}\left(\frac{k_{z,4}k_4^{*}k_{z,2}^{''}}{k_4\left|k_{z,2}\right|^2}\right)\right]}{\left|1-r_{20}^{TM}r_{24}^{TM}e^{-2k_{z,2}^{''}g}\right|^2}\right\} \qquad \text{(S6)}$$

$$q_{\omega,prop}^{1\to 4}\left(\omega,T_1\right)=\frac{\theta\left(\omega,T_1\right)}{\pi^2}\int_0^{k_v}k_r dk_r\left\{\frac{\left[1-\left|r_{20}^{TE}\right|^2-\left|t_{20}^{TE}\right|^2\cdot\mathrm{Re}\left(\frac{k_{z,0}^{*}k_{z,2}^{'}}{\left|k_{z,2}\right|^2}\right)\right]\cdot\left[\left|t_{24}^{TE}\right|^2\cdot\mathrm{Re}\left(\frac{k_{z,4}^{*}k_{z,2}^{'}}{\left|k_{z,4}\right|^2}\right)\right]}{4\left|1-r_{20}^{TE}r_{24}^{TE}e^{2ik_{z,2}^{'}g}\right|^2}+\ldots\right.$$

$$\left.\ldots+\frac{\left[1-\left|r_{20}^{TM}\right|^2-\left|t_{20}^{TM}\right|^2\cdot\mathrm{Re}\left(\frac{k_{z,0}k_0^{*}k_{z,2}^{'}}{k_0\left|k_{z,2}\right|^2}\right)\right]\cdot\left[\left|t_{24}^{TM}\right|^2\cdot\mathrm{Re}\left(\frac{k_{z,4}k_4^{*}k_{z,2}^{'}}{k_4\left|k_{z,2}\right|^2}\right)\right]}{4\left|1-r_{20}^{TM}r_{24}^{TM}e^{2ik_{z,2}^{'}g}\right|^2}\right\} \qquad \text{(S7)}$$

$$q_{\omega,eva}^{1\to4}(\omega,T_1)=\frac{\theta(\omega,T_1)}{\pi^2}\int_{k_v}^{\infty}k_r dk_r e^{-2k_{z,2}''g}\left\{\frac{\left[\mathrm{Im}\left(r_{20}^{TE}\right)-\frac{\left|t_{20}^{TE}\right|^2}{2}\cdot\mathrm{Re}\left(\frac{k_{z,0}^{*}k_{z,2}''}{\left|k_{z,2}\right|^2}\right)\right]\cdot\left[\frac{\left|t_{24}^{TE}\right|^2}{2}\cdot\mathrm{Re}\left(\frac{k_{z,4}^{*}k_{z,2}''}{\left|k_{z,2}\right|^2}\right)\right]}{\left|1-r_{20}^{TE}r_{24}^{TE}e^{-2k_{z,2}''g}\right|^2}+\dots\right.$$

$$\left.\dots+\frac{\left[\mathrm{Im}\left(r_{20}^{TM}\right)-\frac{\left|t_{20}^{TM}\right|^2}{2}\cdot\mathrm{Re}\left(\frac{k_{z,0}k_0^{*}k_{z,2}''}{k_0\left|k_{z,2}\right|^2}\right)\right]\cdot\left[\frac{\left|t_{24}^{TM}\right|^2}{2}\cdot\mathrm{Re}\left(\frac{k_{z,4}k_4^{*}k_{z,2}''}{k_4\left|k_{z,2}\right|^2}\right)\right]}{\left|1-r_{20}^{TM}r_{24}^{TM}e^{-2k_{z,2}''g}\right|^2}\right\} \quad \text{(S8)}$$

where g is the gap distance of the vacuum layer. The combination of equations (S5), (S6), (S7), and (S8) thus provides a set of equations to describe thermal emission from a hot thin-film, which is absorbed by a cold thin-film and a supporting substrate. However, to completely describe the system in Fig. S5, it is also necessary to consider thermal emission from the supporting substrate of the hot emitter.

In order to isolate this contribution, it is possible to make use of the formulation for radiative heat transfer between two semi-infinite media. Despite the presence of thin-films, previous studies [6-8] have proven that the formulation for two semi-infinite media can still be used to calculate the total radiative heat transfer for a hot emitter and cold absorber composed of an arbitrary number of layers assuming the hot and cold side temperatures are uniform across their respective layers. The spectral radiative heat flux in this case will consist of the following,

$$q_{\omega,prop}^{01\to34}(\omega,T_1)=\frac{\theta(\omega,T_1)}{\pi^2}\int_{0}^{k_v}k_r dk_r\left\{\frac{\left[1-\left|r_{20}^{TE}\right|^2\right]\cdot\left[1-\left|r_{24}^{TE}\right|^2\right]}{4\left|1-r_{20}^{TE}r_{24}^{TE}e^{2ik_{z,2}'g}\right|^2}+\frac{\left[1-\left|r_{20}^{TM}\right|^2\right]\cdot\left[1-\left|r_{24}^{TM}\right|^2\right]}{4\left|1-r_{20}^{TM}r_{24}^{TM}e^{2ik_{z,2}'g}\right|^2}\right\} \quad \text{(S9)}$$

$$q_{\omega,eva}^{01\rightarrow 34}(\omega,T_1)=\frac{\theta(\omega,T_1)}{\pi^2}\int_{k_v}^{\infty}k_r dk_r e^{-2k''_{z,2}g}\left\{\frac{\left[\mathrm{Im}\left(r_{20}^{TE}\right)\right]\cdot\left[\mathrm{Im}\left(r_{24}^{TE}\right)\right]}{\left|1-r_{20}^{TE}r_{24}^{TE}e^{-2k''_{z,2}g}\right|^2}+\frac{\left[\mathrm{Im}\left(r_{20}^{TM}\right)\right]\cdot\left[\mathrm{Im}\left(r_{24}^{TM}\right)\right]}{\left|1-r_{20}^{TM}r_{24}^{TM}e^{-2k''_{z,2}g}\right|^2}\right\} \quad \text{(S10)}$$

where it is assumed the temperature of substrate 0 is equal to the temperature of film 1. The reflection coefficients represent the total reflectance of the thin-film and substrate for the hot and cold sides. By combining Eqs. (S5) - (S10), the emission from thin film 1 to substrate 3 and 4 can be calculated. We now have a complete set of equations to describe radiative heat transport for a system composed of a single thin-film supported by a substrate on both the hot and cold sides. This formulation will serve as the foundation in the manuscript to explore thin-film morphological effects on near-field radiative heat transfer using dielectrics and metals.

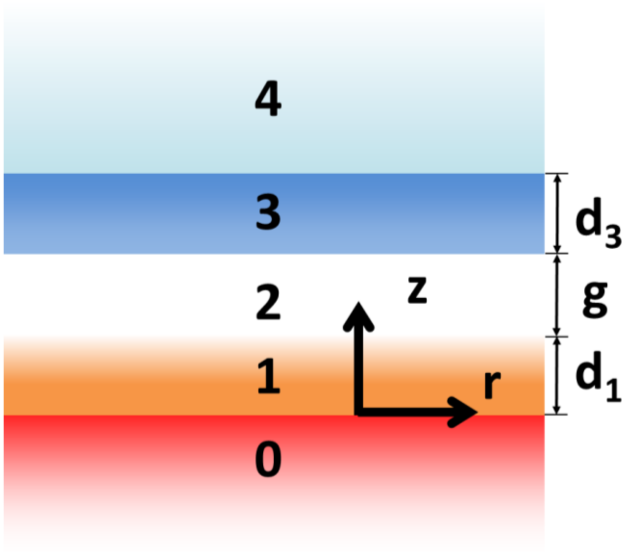

Fig. S5 The schematic of near-fild radiation. Materials 0 and 4 are semi-infinite substrates. Materials 1 and 3 are the thin films. Material 2 is vacuum. Z is the direction perpendicular to all substrates, and r is the in-plane direction.

Section III. The Net Generation Rate through Surface Defect Process

In the neutral regions of a p-n junction, minority carriers are driven by the diffusion force so

that the drift-diffusion equation in p-type neutral region can be simplified to $I_e = qD_e\nabla n$, where the I_e is the electron current density, D_e is the diffusivity of electron, and n is the electron concentration. [9-10] Under these assumptions, the minority carrier concentration profile in one dimension can be found by solving

$$-D_e\frac{d^2n'}{dx^2} = [G_{rad} - R_{rad}] + \left[G_{Auger} - R_{Auger}\right] \approx [G_{rad} - R_{rad}] - \frac{n'}{\tau_{Auger}} \qquad \text{(S11)}$$

where $\tau_{Auger} \approx \frac{1}{(k_{eeh}n_o+k_{ehh}p_o)(n_o+p_o)}$ is the Auger lifetime, k_{eeh} and k_{ehh} are the Auger rate constants, $n' = n - n_o$, n is the electron non-equilibrium concentration, n_o and p_o are the electron and hole equilibrium concentration respectively, the G_{rad} and G_{Auger} are the free carrier generation rates for radiative and Auger processes respectively, and the R_{rad} and R_{Auger} are the free carrier recombination rates for radiative and Auger processes respectively.

To solve this equation, the generation and recombination rate of the radiative process are calculated by rigorously solving Maxwell equations under various chemical potential and applied voltages. Combining the boundary conditions, $D_e\frac{dn'}{dx} = S_pn'$ at $x = -l_p$ and $n' \approx n_o(\exp\left(\frac{qV}{k_bT}\right) - 1)$ at $x = -x_p$ where the S_p and S_n are the surface process velocity, [9-10] the minority carrier can be solved. Finally, the net surface generation rate can be calculated using $S_pn'(x = -l_p)$, and the net surface generation rate can also be solved in the similar method in n-type neutral region. The dimensions of the pn junction are shown in Fig. S6.

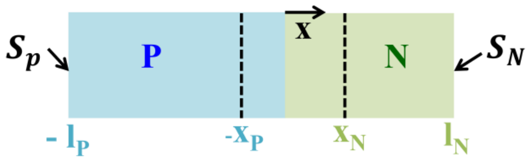

Fig. S6 The schematic of a pn junction. [-x_P, x_N] is the space charge region, and [-l_P,-x_P] and [x_N, l_N] are the neutral regions in p-type and n-type respectively.

Reference:

[1] Long, L. L., Querry, M. R., Bell, R. J., and Alexander, R. W. Optical properties of calcite and Gypsum in crystalline and powdered form in the infrared and far-infrared, *Infrared Phys*. **34**, 191-201 (1993).

[2] Adachi, S. Optical dispersion relations for GaP, GaAs, GaSb, InP, InAs, InSb, Al x Ga1-x As, and In1-x Ga x As y P 1-y, *J. Appl. Phys*. **66**, 6030 (1989).

[3] Moss, T. S., Smith, S. D. and Hawkins, T. D. F. Absorption and dispersion of Indium Antimonide, *Proceedings of the Physical Society, Section B*, **70**, 776-784 (1957).

[4] Tong, J.K. et al. Thin-film 'thermal well' emitters and absorbers for high-efficiency thermophotovoltaics, *Scientific Reports*, **5**, 10661, 2015.

[5] Boriskna, S.V. et al. Enhancement and tunability of near-field radiative heat transfer mediated by surface plasmon polaritons in thin plasmonic films, *Photonics*, **2**, 659, 2015.

[6] Biehs, S.-A., Tschikin, M., Messina, R., and Ben-Abdallah, P. Super-Plankian near-field thermal emission with phonon-polaritonic hyperbolic metamaterials. Appl. Phys. Lett. 102, 131106 (2013).

[7] Tschikin, M., Ben-Abdallah, P., and Biehs, S. A. Coherent thermal conductance of 1-D photonic crystals. Physics Letters A 376, 3462-3465 (2012).

[8] Francoeur, M., Mengüç, M.P., and Vaillon, R. Solution of near-field thermal radiation in one-dimensional layered media using dyadic Green's functions and the scattering matrix method. *Journal of Quantitative Spectroscopy and Radiative Transfer* **110**, 2002-2018 (2009).

[9] Sze, S. M. and Ng, K. K. *Physics of Semiconductor Devices, 3rd Edition.;* John Wiley & Sons, Inc.; NJ (2007).

[10] del Alamo, J. A. *Integrated Microelectronic Devices: Physics and Modeling.* Prentice Hall (2013).
